# Dipeptidyl Peptidase IV as a Muscle Myokine

**DOI:** 10.3389/fphys.2020.00148

**Published:** 2020-03-03

**Authors:** Heidi A. Kluess

**Affiliations:** School of Kinesiology, Auburn University, Auburn, AL, United States

**Keywords:** peptidase, muscle, secretome, exercise, whey protein, metalloproteases

## Abstract

Dipeptidyl peptidase IV (DPP-IV) is a unique serine protease that exists in a membrane bound state and in a soluble state in most tissues in the body. DPP-IV has multiple targets including cytokines, neuropeptides, and incretin hormones, and plays an important role in health and disease. Recent work suggests that skeletal muscle releases DPP-IV as a myokine and participates in control of muscle blood flow. However, few of the functions of DPP-IV as a myokine have been investigated to date and there is a poor understanding about what causes DPP-IV to be released from muscle.

## Introduction

Dipeptidyl Peptidase IV (DPP-IV) is a serine protease that cleaves a variety of proteins that contain the X-Pro and X-Ala dipeptides including incretin hormones, many neuropeptides, and some cytokines (Mulvihill and Drucker, 2014). DPP-IV is present in a membrane bound form in most cells the body including the gut, kidneys, on the surface of T-cells, on the endothelial layer of arteries and arterioles and on skeletal muscle. The function of membrane bound DPP-IV in T-cells is well characterized (Boonacker and Van Noorden, 2003), but its action in other tissues is less well understood.

Dipeptidyl Peptidase IV can also exist as an active enzyme in a soluble form in the interstitial space and in the blood. The blood contains a concentration of soluble DPP-IV in the plasma at activities ranging from 12.5 to 42 U/L in the general population (Durinx et al., 2001) and from 13.6 to 73 U/L in a healthy younger group (Neidert et al., 2016c). The origin of blood DPP-IV is believed to be all of the sources of DPP-IV including the gut, kidneys, muscle, T-cells and fat (Mentlein, 1999). The most well understood purpose of DPP-IV in the plasma is truncating incretin hormones such as GLP-1 into a non-usable form and thus, altering insulin release (Batterham and Bloom, 2003). Inhibiting DPP-IV is a pharmacological treatment for diabetes and results in significant improvement in bioactive GLP-1 and insulin release (Lambeir et al., 2003). Additional benefits of DPP-IV inhibition are a reduction in TNF-alpha (Akarte et al., 2012) and a reduction in cytokines like IL-2, IL-6, and IL-10 in some studies (Klemann et al., 2016). DPP-IV inhibition also improves exercise capacity and mitochondrial function in mice with heart failure (Takada et al., 2016) and improves liver function in diabetic mice (Tanimura et al., 2019). On the negative side, DPP-IV inhibitors can cause severe joint/muscle pain and neuropeptide Y-mediated hypertension in some people (Klemann et al., 2016).

Despite the widespread presence of DPP-IV in the body, the actual functions of DPP-IV in most tissues is poorly understood. The purpose of this review is to investigate the current knowledge of DPP-IV in and around muscle and the current knowledge of the actions of DPP-IV on the muscle.

## DPP-IV and Skeletal Muscle Blood Flow

Neuropeptide Y is a powerful vasoconstrictor that plays an important role in skeletal muscle blood flow regulation in humans (Zukowska et al., 2003), dogs (Buckwalter et al., 2004, 2005), and rodents (Jackson et al., 2005a; Evanson et al., 2011; Al-Khazraji et al., 2015). One of the most well understood purpose of DPP-IV is the truncation the sympathetic vasoconstrictor, neuropeptide Y, into a form that does not result in Y1-receptor mediated vasoconstriction. DPP-IV is bound to the endothelium (Lewandowski et al., 1998; Kitlinska et al., 2002; Pala et al., 2012) and the smooth muscle (Chung et al., 2010; Evanson et al., 2011, 2012) of arterioles. DPP-IV is also found bound to the smooth muscle membrane of the abluminal surface of skeletal muscle arterioles (Chung et al., 2010), where it truncates approximately 40% of the neuropeptide Y (NPY_1__–__36_) released from the sympathetic neurons, to become NPY_3__–__36_ (Evanson et al., 2011, 2012). This results in reduced NPY-mediated vasoconstriction and, in turn, improved blood flow to the skeletal muscle (Jackson et al., 2005a; Dubinion et al., 2006; Neidert et al., 2018). The effect of DPP-IV on NPY may be greater in females compared to males. Jackson et al. (2005b) saw a decrease of 34% in vascular conductance after blocking DPP-IV and aminopeptidase P. Work from my lab (Evanson et al., 2012) explored the idea that estrogen may be the cause of this difference between males and females with regard to DPP-IV. Sixty days of being ovariectomized with or without estrogen replacement did not change DPP-IV activity or alter neuropeptide Y degradation (Evanson et al., 2012). However, Younan et al. (2019) investigated the role of DPP-IV in liver inflammation after ovariectomy in rats. They found that DPP-IV inhibition reduced liver inflammation, but this study did not measure DPP-IV activity directly. In a recent study, we looked at normally menstruating women versus post-menopausal women and saw no difference in plasma DPP-IV activity (Kluess et al., 2019), demonstrating that the effect of estrogen appears to be consistent across species.

## Evidence That DPP-IV is Released From the Muscle

Although plasma or serum DPP-IV is a widely used sampling location to measure DPP-IV, it is difficult to attribute plasma DPP-IV exclusively to muscle released DPP-IV. However, in a group of 111 people we found a positive relationship between plasma DPP-IV and lean mass measured by Dual X-ray Absorptiometry. This relationship explained about 14% of the variation, suggesting that in young healthy people a portion of the DPP-IV in the plasma likely does come from the muscle (Neidert et al., 2016c).

The evidence that muscle can release DPP-IV is quite recent. Raschke et al. (2013) showed that DPP-IV is released by skeletal muscle cell culture during differentiation. Neidert et al. (2016b) demonstrated that DPP-IV was released from skeletal muscle cell culture with the application of whey protein isolates, but only DPP-IV mRNA increased with exercise-like modulators such as caffeine, and hydrogen peroxide. Whey protein also increased DPP-IV activity in the bathing medium from an intact skeletal muscle *in situ* (Neidert et al., 2018). However, in this model we could not distinguish between skeletal muscle-released and smooth muscle-released DPP-IV. There are no studies to date investigating the release of DPP-IV from contracting muscle. It is very difficult to do this work *in vivo* because you cannot distinguish muscle released DPP-IV from DPP-IV released from other sources.

## Mechanism of Release of DPP-IV From the Muscle

**FIGURE 1:**
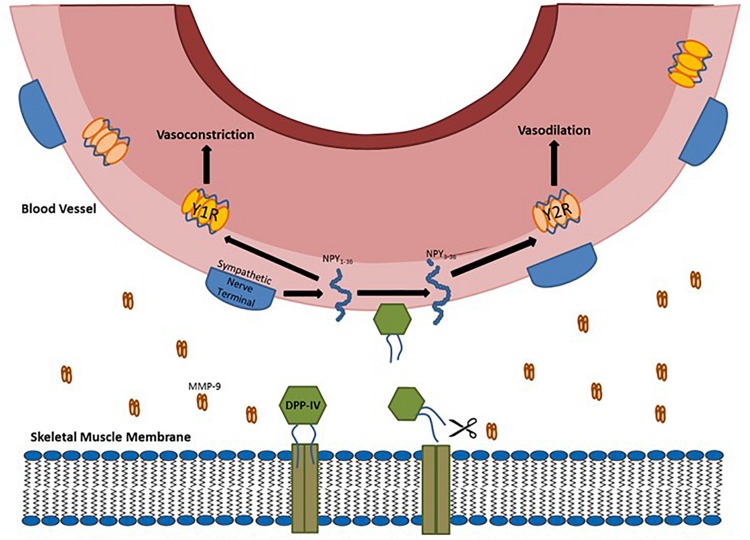
Diagram of DPP-IV being shed from the membrane by metalloproteases (MMP) and the soluble DPP-IV cleaving full length NPY into NPY3-36.

The process of DPP-IV release from the muscle is a multi-step process. DPP-IV starts bound to the endoplasmic reticulum (Klemann et al., 2016). It then migrates to the muscle cell surface and remains in a functional, but membrane-bound state. Hooper et al. (1997a, b) first identified that phosphatidylinositol-specific phospholipase C could cause release of membrane dipeptidases. The idea that DPP-IV could be shed from the membrane by extracellular proteases was further refined by Röhrborn et al. (2014). They showed that the metalloproteases 1, 2, and 14 are involved in shedding DPP-IV from the membranes of smooth muscle and adipocytes. Neidert et al. (2016b) demonstrated that DPP-IV shedding occurs in skeletal muscle myocyte cell culture using whey protein (a source of metalloproteases) (Raulo et al., 2002; Lubetzky et al., 2010). This hypothesis was also confirmed using specific inhibitors for metalloprotease 2 and 9 and a general protease inhibitor. In a follow-up study (Neidert et al., 2018), we used whey protein to stimulate shedding of DPP-IV from skeletal muscle *in situ* and showed an increase in DPP-IV activity in the muscle bathing medium and an increase in skeletal muscle arteriolar diameter. This effect was inhibited by adding a DPP-IV inhibitor to the media bathing the preparation. This finding suggested that one reason for DPP-IV release from the membrane may be the reduction in neuropeptide Y-mediated vasoconstriction (see Figure 1 for a diagram of the possible mechanism). DPP-IV may also be involved in shortening the half-life of some cytokines such as IL-6 (Ikeda et al., 2013), but there is no conclusive evidence for this. DPP-IV is known to target stromal cell derived factor 1α (Christopherson et al., 2002) and therefore may be involved in targeting T-cells to damaged skeletal muscle. To date this possibility has not been investigated.

## Exercise and Exercise Training Related Changes in DPP-IV

One of the challenges of measuring DPP-IV changes with exercise or exercise training is the ability to sample DPP-IV. The easiest method is to take a blood sample and measure DPP-IV in the plasma or serum. The downside of this is that the source of the change in DPP-IV may be a variety of sources such as adipose tissue, muscle, immune cells and other areas of the body influenced by exercise. A further complication in the measurement of DPP-IV activity in the plasma is it is so well buffered. For example, it is well described that DPP-IV in the blood does not change with feeding (Meneilly et al., 2000; Ryskjaer et al., 2006; Neidert et al., 2016a) or acute exercise (Neidert et al., 2016b). However, there are several studies that have attempted to measure DPP-IV changes in the muscle or plasma with acute exercise. In anesthetized rats, the gastrocnemius muscle was electrically stimulated to create four sets of dynamic plantar flexions. Immediately after they were either gavage fed whey protein or no whey protein. From the muscles harvested from the rats, we found an increase in DPP-IV mRNA only when exercise was combined with whey protein feeding (Neidert et al., 2016b). In humans, taking whey protein prior to a maximal exercise test also resulted in elevated DPP-IV in the plasma, but the maximal exercise test alone failed to increase plasma DPP-IV (Kluess and Neidert, 2018). We also performed a resistance exercise protocol designed to cause muscle soreness in the leg muscles. This protocol did cause muscle soreness, but failed to increase serum DPP-IV (Neidert et al., 2016b). We suspected the leg exercise protocols failed to increase DPP-IV because of buffering by other sources of DPP-IV from the legs to the sampling location in the arm. Studies that sample close to the source of the exercising muscles are needed.

There are limited number of studies looking at exercise and DPP-IV inhibition. Takada et al. (2016) treated mice with heart failure with a DPP-IV inhibitor for 4 weeks and found an improvement in peak VO_2_. This effect may have something to do with GLP-1 because a GLP-1 inhibitor abolished the improvement in VO_2_peak. Further, they saw improved mitochondrial function and fiber type shifts from IIB to I with DPP-IV inhibition. This same positive effect of DPP-IV inhibition has not been seen in humans with heart failure (Bassi and Fonarow, 2018) and in fact, may be harmful in some people with heart failure (Clifton, 2014; Lourenco et al., 2017). Considering the effect that DPP-IV has on muscle blood flow and muscle function, more studies investigating the muscle effects of DPP-IV inhibition are warranted.

There is little literature on the changes in DPP-IV with exercise training. Tanimura et al. (2019) showed improved blood triglycerides and reduced hepatic lipid accumulation in diabetic mice on a high fat diet with exercise training and a DPP-IV inhibitor. There are two studies in humans that measure plasma DPP-IV after exercise training. Lee et al. (2015) studied adolescents with type 2 diabetes and measured a variety of outcomes after 12 weeks of high or low intensity exercise training. The high intensity training group lost weight and saw a decrease in insulin. Both training groups saw an improvement in VO_2_max, higher GLP-1 and a reduction in serum DPP-IV. In obese adults with metabolic syndrome, Malin et al. (2013) saw lower plasma DPP-IV and improved insulin sensitivity with a decrease in body weight and fat mass following 12 weeks of supervised exercise. To date there are no studies in healthy people regarding DPP-IV changes with exercise training.

## Conclusion

Dipeptidyl peptidase IV is an enzyme with broad effects throughout the body. It is released by a variety of tissues including muscle. In the muscle, DPP-IV is released from the membrane by metalloproteases in the interstitial space. The effects of DPP-IV on muscle include the reduction in neuropeptide Y-mediated vasoconstriction to increase muscle blood flow. Other possible effects of DPP-IV are uninvestigated to date. DPP-IV has many targets that may influence muscle function and many research questions remain.

## Author Contributions

The author confirms being the sole contributor of this work and has approved it for publication.

## Conflict of Interest

The author declares that the research was conducted in the absence of any commercial or financial relationships that could be construed as a potential conflict of interest.
